# Diseases of complement dysregulation—an overview

**DOI:** 10.1007/s00281-017-0663-8

**Published:** 2018-01-11

**Authors:** Edwin K. S. Wong, David Kavanagh

**Affiliations:** 1 The National Renal Complement Therapeutics Centre, aHUS Service, Building 26, Royal Victoria Infirmary, Queen Victoria Road, Newcastle upon Tyne, NE1 4LP UK; 2 Institute of Cellular Medicine, Newcastle University, Newcastle upon Tyne, UK

**Keywords:** Complement, C3G, aHUS, PNH

## Abstract

Atypical hemolytic uremic syndrome (aHUS), C3 glomerulopathy (C3G), and paroxysmal nocturnal hemoglobinuria (PNH) are prototypical disorders of complement dysregulation. Although complement overactivation is common to all, cell surface alternative pathway dysregulation (aHUS), fluid phase alternative pathway dysregulation (C3G), or terminal pathway dysregulation (PNH) predominates resulting in the very different phenotypes seen in these diseases. The mechanism underlying the dysregulation also varies with predominant acquired autoimmune (C3G), somatic mutations (PNH), or inherited germline mutations (aHUS) predisposing to disease. Eculizumab has revolutionized the treatment of PNH and aHUS although has been less successful in C3G. With the next generation of complement therapeutic in late stage development, these archetypal complement diseases will provide the initial targets.

## Introduction

Atypical hemolytic uremic syndrome (aHUS), C3 glomerulopathy (C3G), and paroxysmal nocturnal hemoglobinuria (PNH) serve as exemplars of the mechanisms by which complement dysregulation may cause disease. In this review, we shall compare and contrast the underlying pathophysiological mechanisms and the response to treatments.

## Atypical HUS

### Classification of atypical hemolytic uremic syndrome

**Fig. 1:**
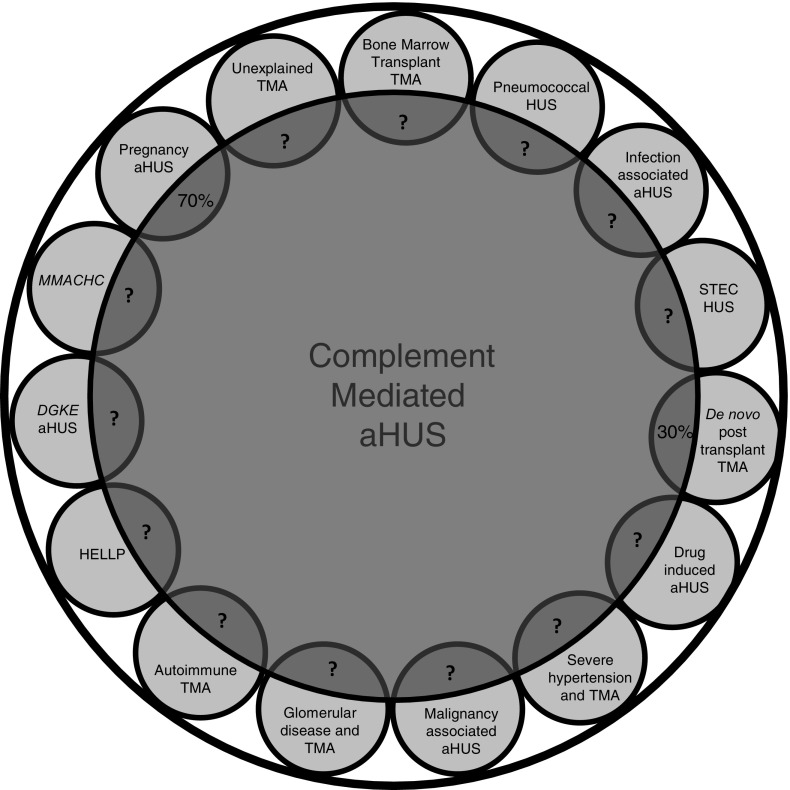
The role of complement in thrombotic microangiopathies. A mutation or autoantibody resulting in complement dysregulation predisposes to complement-mediated aHUS. Complement-mediated aHUS frequently only manifests upon exposure to an environmental trigger, which can include other causes of TMA. In some TMAs, a high proportion of individuals carry a mutation (e.g., pregnancy associated aHUS, ~ 70%, and de novo post-transplant TMA, ~ 30%) but in others the incidence of mutations is unknown or low (e.g., STEC-HUS). In other TMAs, complement activation may be seen in vivo but whether it plays a role as a disease modifier or is simply a bystander is yet to be clarified

The last 20 years has seen striking advances in our understanding of the molecular mechanisms underlying thrombotic microangiopathies (TMAs) and with this has come a complex and rapidly evolving nomenclature [1]. Historically, TMAs were categorized on clinical findings: HUS for renal dominant disease, thrombotic thrombocytopenic purpura (TTP) for predominant neurological involvement. Subsequently, TTP was defined by severe ADAMTS13 deficiency; HUS caused by shiga toxin-producing *Escherichia coli* (STEC) defined as STEC-HUS, with aHUS broadly used for all other causes of TMA. With the discovery of genetic and acquired complement dysregulation in a proportion of patients with aHUS, the term complement-mediated aHUS was used to refer to this subgroup. When reviewing historical literature, “aHUS” may refer specifically to complement-mediated TMA, or be more loosely applied to any TMA that is not TTP or STEC-HUS (reviewed [1]). In this review, we use the term complement-mediated aHUS when the etiology is defined as such, and use aHUS where etiology is ill defined. Current classifications describe acquired primary TMAs, inherited primary TMAs, secondary TMAs, and infection-associated TMAs (Table 1) although it should be borne in mind that underlying complement genetic predispositions often require a secondary trigger for TMA to manifest. The role of complement in secondary TMAs and infection associated TMA is yet to be defined (Fig. 1).

**Table 1:** Classification of thrombotic microangiopathies

Primary TMA: hereditary
aHUS with complement gene mutation
(*CFH*; *CFI*; *CFB*; *C3*; *CD46*; *CFHR1* hybrid)
TTP with *ADAMTS13* mutation
MMACHC TMA
DGKE TMA
Primary TMA: hereditary
aHUS with complement autoantibodies
(anti-FH; anti-FI)
TTP with ADAMTS13 autoantibody
Secondary TMAs
TMA with glomerular disease
(FSGS; IgAN, C3G/MPGN, MN, AAV)
Malignancy associated TMA
Drug induced TMA
Direct toxicity (interferon B; bevacizumab)
Immune mediated damage (e.g., quinine)
TMA with autoimmune conditions
(SLE, SRC, CAPS)
De novo TMA after solid organ transplant
HELLP
Infection associated TMA
STEC-HUS
Pneumococcal HUS
HIV associated aHUS
Other

*AAV* ANCA (anti-neutrophil cytoplasmic antibody) associated vasculitis; *ADAMTS13* a disintegrin and metalloproteinase with a thrombospondin type 1 motif, member 13; *aHUS* atypical hemolytic uremic syndrome; *C3G* C3 glomerulopathy; *CAPS* catastrophic antiphospholipid syndrome; MMACHC Methylmalonic aciduria and homocystinuria, *cblC* type; *DGKE* gene encoding diacylglycerol kinase Ɛ; *FH* factor H; *FI* factor I, *FSGS* focal segmental glomerulosclerosis; *HELLP* syndrome of hemolysis, elevated liver enzymes, and low platelets; *HIV* human immunodeficiency virus; *HUS* hemolytic uraemic syndrome; *IgAN* IgA nephropathy; *MN* membranous nephropathy; *MPGN* membranoproliferative glomerulonephritis; *SLE* systemic lupus erythematosus; *SRC* scleroderma renal crisis; *STEC*, shiga toxin-producing *Escherichia coli*; *TMA* thrombotic microangiopathy; *TTP* thrombotic thrombocytopenic purpura

## Pathology

The pathological findings seen in complement-mediated aHUS reflect tissue responses to endothelial injury: endothelial swelling and mesangiolysis in active lesions, double contours of the basement membrane in chronic lesions (reviewed [2]). The absence of overt platelet fibrin thrombosis from renal biopsies of TMA has recently led to a suggested reclassification to microangiopathy +/− thrombosis [2].

### Inherited primary complement-mediated aHUS

First described in 1998 by Warwicker et al. [3], mutations in factor H (*CFH*) are the commonest cause of inherited complement-mediated aHUS, accounting for around 25% of cases [4]. Factor H (FH) is the most important fluid-phase regulator of the alternative pathway (AP) of complement. FH is composed of 20 complement control protein modules (CCPs), also known as short consensus repeats (SCRs). The four N-terminal CCPs (CCPs 1–4) mediate the complement regulatory functions of the protein by competing with factor B for C3b binding, accelerating the decay of the C3 convertase into its components, and acting as a cofactor for factor I-mediated proteolytic inactivation of C3b [5, 6]. The vast majority of *CFH* mutations seen in complement-mediated aHUS do not occur in this region, but instead in the C terminal domains (CCP 19–20) [4]. It is this region which mediates FH self-surface binding via its interaction with C3b, sialic acid, and glycosaminoglycans [7, 8]. In complement-mediated aHUS, the mutations are usually heterozygous, do not result in a quantitative deficiency of FH but instead have variable consequences on binding to GAGs, sialic acid, and C3b which impairs cell surface complement regulation [9, 10] (reviewed^4^).

In addition to point mutations, its location in the RCA cluster makes *CFH* particularly prone to genomic rearrangements. This is an area of the genome that arose from several large genomic duplications, and these low copy repeats can cause genome instability in this region. The *CFH* mutations S1191L, V1197A, and combined S1191L/V1197A arose through gene conversion between *CFHR1* and *CFH* [11]. A hybrid (fusion) gene comprising the 21 N-terminal exons of *CFH* and the 2 C terminal exons of *CFHR1* was demonstrated to have arisen through nonallelic homologous recombination and resulted in complement-mediated aHUS [12]. More recently, several other hybrid genes consisting of the N-terminal exons of *CFH* and the 5 C-terminal exons of *CFHR3* have been reported [13, 14]. As with C-terminal point mutations in *CFH*, these hybrid genes also result in loss of cell surface complement regulation.

### Membrane cofactor protein

Membrane cofactor protein (MCP; CD46) is a surface-bound complement regulatory protein that acts as a cofactor for the factor I (FI) mediated cleavage of C3b and C4b that are deposited on host cells [15]. Mutations in *CD46* are the second commonest cause of complement-mediated aHUS accounting for around 15% of patients.

The majority of mutations are found in the extracellular domains of CD46 that are responsible for C3b and C4b binding. Unlike *CFH*, most *CD46* mutations result in a quantitative defect in CD46 (~ 75%) [4].

### Complement factor I

Complement factor I is a serum serine protease, which functions as a critical mediator of complement regulation by cleaving C3b and C4b in the presence of its cofactors (FH for C3b; C4BP (C4b binding protein) for C4b, CD46 and CR1 (complement receptor 1) for both [4].

Around 10% of complement-mediated aHUS is predisposed to by mutations in the FI gene (*CFI*) [16]. The *CFI* mutations described in complement-mediated aHUS are all heterozygous.

### Complement C3

C3 is the central component of the complement cascade. C3 is cleaved to form the anaphylatoxin C3a and C3b, which is highly reactive and can bind to cell surfaces via its reactive thioester. C3b can then interact with factor B (FB) in the presence of factor D to form the alternative pathway convertase introducing a positive-feedback loop.

Mutations in *C3* account for around 2–10% of complement-mediated aHUS. The mutations in *C3* linked to complement-mediated aHUS result in complement over activation by either (1) preventing complement regulators binding to C3 and inactivating it or by binding to FB with greater affinity [17–19]. These mutations result in increased complement activation on platelets and glomerular endothelium.

### Complement factor B

Factor B carries the catalytic site of the complement AP convertase (C3bBb). Mutations in *CFB* are very rare in complement-mediated aHUS. As with *C3* mutations, complement over activation occurs by impaired complement regulation or increased convertase formation [20]. These mutations have been demonstrated to increase complement deposition on endothelial cells.

### Thrombomodulin

Thrombomodulin (THBD) plays a key role in regulating clot formation by the activation of protein C by thrombin and enhancing thrombin-mediated activation of plasma procarboxypeptidase B (CPB2), an inhibitor of fibrinolysis. Procarboxypeptidase B also inactivates complement-derived anaphylatoxins C3a and C5a. THBD has also been suggested to have a role in the regulation of the AP by accelerating FI-mediated inactivation of C3b [21].

Rare genetic variants have been described in some aHUS cohorts but their causality remains to be established [21].

### Common genetic susceptibility factors

In addition to rare mutations, a number of common single nucleotide polymorphisms (SNPs) in *CD46* and *CFH* have been associated with complement-mediated aHUS*.* A *CD46* haplotype (*CD46*_ggaac_) block has been associated with a two- to threefold increased risk of complement-mediated aHUS [22–24]. This encompasses 2 SNPs in the promoter region of CD46, and reporter gene assays have suggested that this haplotype reduces transcriptional activity by 25% albeit without decreased CD46 cell surface expression in vivo [24, 25]. A *CFH* haplotype (*CFH*-H3; tgtgt) has been shown to increase the risk of complement-mediated aHUS two- to fourfold [22, 26]. This risk haplotype contains a SNP *CFH*-Val_62_ which has a subtle decrease in cofactor activity compared to the protective variant [27, 28].

### Acquired primary complement-mediated aHUS

Acquired defects in complement regulation have been seen in the form of autoantibodies to FH. First reported in 2005, these account for ~ 10% of complement-mediated aHUS [29]. It predominantly presents in childhood, frequently with a gastrointestinal prodrome [30].

There is a strong association with a homozygous deletion of *CFHR3* and *CFHR1*, which encodes complement factor H-related proteins (FHR) 3 and 1 [31]. Subsequently, FHR1 deficiency resulting from point mutations in *CFHR1* or from a deletion incorporating *CFHR1* and *CFHR4* has been reported in individuals with FH autoantibody-mediated aHUS [32]. Although this suggests a key role in the deficiency of FHR1 in the generation of FH autoantibodies, the mechanism remains obscure and several patients have been reported with factor H autoantibodies in the absence of the *CFHR1/CFHR3* deletion.

The majority of autoantibodies bind to the C-terminal domain of FH, thus mimicking the defects see in the inherited form. The antibodies have been shown to reduce binding to C3b and other C3 fragments [33, 34]. They perturb FH-mediated cell surface protection and in some individuals the autoantibodies also impair cofactor activity or decay accelerating activity [33, 34].

Autoantibodies against FI have also been reported, but are rare and their functional relevance remains to be established [35].

### Clinical presentation and outcome

The incidence of aHUS is ~ 0.4/million population [36]. Hemolysis and ischemic organ injury, predominantly in the kidney, define the clinical presentation of aHUS [1]. Extra-renal manifestations (cardiac, ocular, pancreatic) are reported although it is not known whether they are a direct consequence of the TMA, a direct effect of complement activation, or relate to the associated severe hypertension and uremia [4].

Historically, the prognosis for patients with aHUS was poor and in the pre-eculizumab era at 3–5 years after onset, 36–48% [22, 37] of children and 64–67%, [37] of adults had died or reached ESRD. The underlying genetic cause predicted the outcome of disease with those carrying *CD46* mutations having the best prognosis (3 year renal survival 94%). In individuals with *CFH* mutations, up to 77% of patients had developed ESRD or had died at 3–5 years. Only 30–40% of individuals with *CFI* and *C3* mutations will be alive with native kidney function at 3–5 years, [4, 37]. In those with FH-autoantibodies, 36.5–63% die or reach ESRD over a similar timescale.

The outcome following renal transplantation was also poor and again the outcome was predicted largely by the underlying genetic abnormality. The overall recurrence was 68% and 5-year death-censored graft survival 51% with highest risk associated with *CFH*, *CFB*, and *C3* mutations and the lowest with *CD46* mutations. [38, 39]

### Disease penetrance

The genetic mutations seen in complement-mediated aHUS are not causative but are instead predisposing, suggesting that additional genetic and environmental modifiers are important. Penetrance of disease is age related and has been reported to be as high as 64% by the age of 70 for individuals carrying a single genetic mutation [2]. A small proportion of aHUS patients (~ 3%) will have more than one mutation with increased penetrance per additional mutation [40]. Risk haplotypes have also been shown to increase disease penetrance. Even where an individual has multiple genetic risk factors, a trigger is frequently required (e.g., infections [41], pregnancy [42]) to unmask a latent complement defect. These triggers usually activate complement: complement activation is the normal physiological response to infection and occurs in the placenta in normal pregnancy.

### Eculizumab in aHUS

The elucidation of the role of complement in disease provided the rationale for the use of eculizumab in complement-mediated aHUS. Eculizumab, a recombinant humanized monoclonal antibody directed against C5, blocks the cleavage of C5 into its effector components C5a and C5b. Landmark studies in primary aHUS published in 2013 demonstrated its efficacy which have been replicated in subsequent extension studies [43], prospective (non-randomized) studies [44, 45], and cohort analysis [36]. The role of eculizumab in secondary aHUS remains to be established (reviewed [46]).

In individuals requiring a renal transplant with a diagnosis of primary complement-mediated aHUS, the high recurrence rate following transplantation necessities pre-emptive eculizumab [2, 39].

The optimal length of treatment with eculizumab for individuals presenting with complement-mediated aHUS is unclear although the current license for eculizumab is for lifelong treatment; eculizumab withdrawal has been reported in a large series of aHUS patients with relapse reported in around one third of patients, all carrying complement mutations [47]. In those patients experiencing a relapse post withdrawal, rapid reintroduction of complement inhibition normalized the renal function. This suggests that a disease-driven intermittent regime could be used although prospective trials are required.

### Eculizumab non-responsive aHUS

There is no biomarker currently that will confirm the diagnosis of a primary complement-mediated aHUS in the acute setting, and the diagnosis is therefore one of exclusion. As early initiation of eculizumab has been shown to lead to better outcomes, treatment is often commenced in patients with suspected primary complement-mediated aHUS, and discontinued if an alternative etiology is subsequently identified. With the increasing use of eculizumab in clinical practice, it has become apparent that there are subgroups of aHUS that do not respond to eculizumab. In the recent pediatric trial, Greenbaum et al. highlighted that for those with a rare genetic variant in the complement system or an autoantibody to FH, all had an improvement in estimated glomerular filtration rate (eGFR), while 27% of individuals without an identified complement abnormality failed to show an improvement [45]. It is not clear whether this represents late presentation of disease or true non-response.

More recently, individuals presenting with a TMA with failure to respond to eculizumab have been demonstrated to have genetic variants in the non-complement genes *DGKE* [48], *INF2* [49], and *MMACHC* [50].

### DGKE

Diacylglycerol kinase epsilon (DGKE) is a lipid kinase that catalyzes the phosphorylation of diacylglycerol substrates (DAGs) to phosphatidic acid. Recessive mutations causing aHUS were first reported in 2013 [48]. The exact mechanisms resulting in the TMA are yet to be fully elucidated; however, loss of DGKE function results in enhanced signaling through arachidonic acid-containing DAGs (AADAGs) and enhanced activation of PKC (protein kinase C). In the endothelium, PKC activation results in upregulation of prothrombotic factors and the downregulation of VEGFR2 signaling and these may play a role.

Only a small number of cases have been published, but it appears to present aged < 1 year and commonly results in progressive chronic kidney disease (CKD) and ESRF [37]. There is insufficient evidence to determine optimal management; there are reports of both response and non-response to eculizumab. Concomitant mutations in complement genes have been reported. Genetic pleiotropy is seen: DGKE mutations have also been associated with mesangioproliferative glomerulonephritis (MPGN) [51] .

### Inverted formin 2

Mutations in inverted formin 2 (*INF2*) have recently been reported in families with TMAs which was non-responsive to eculizumab [49]. INF2 is a ubiquitously expressed formin protein which accelerates actin polymerization and depolymerization, thus regulating a range of cytoskeleton dependent cellular functions including the secretory pathway. As with *DGKE*, genetic pleiotropism is also seen with most individuals with *INF2* mutations presenting with focal segmental glomerulosclerosis (FSGS) and nephrotic syndrome. It remains to be seen whether this is a primary aHUS or secondary phenomenon in association with FSGS.

### Methylmalonic aciduria and homocystinuria, cobalamin C (cblC) type

Homozygous or compound heterozygous mutations in the *MMACHC* gene result in a disorder of cobalamin (cbl; vitamin B12) metabolism that causes aHUS. Although the pathophysiologic mechanisms that result in endothelial damage are unclear, metabolic therapy with hydroxycobalamin is very effective at preventing disease. It appears that MMACHC-mediated aHUS is complement independent as the small number of published reports of eculizumab use describe non-response [50].

### Polymorphisms in C5 and the use of Coversin to treat TMA

A rare polymorphism in *C5* (p.R885H) has been reported in the Japanese population which prevented eculizumab binding [52]. More recently, a European with a functionally significant *CFH* mutation (p.D1119G) and a TMA post-bone marrow transplant was shown to carry this SNP preventing the use of eculizumab [53]. In this case, an alternative C5 inhibitor, Coversin, was used. This is a recombinant protein derived from the tick, *Ornithodoros moubata*. As this binds to a different epitope on C5, this completely blocked the terminal pathway and there appeared to be a clinical response although there was a limited supply of the drug and the patient died [53].

## C3 Glomerulopathy

### Introduction

C3 glomerulopathy (C3G) is a recently identified pathological entity describing a group of diseases in which uncontrolled complement activation can lead to complement deposition within the glomerulus [54]. Historically, uncontrolled complement activation has been associated with the disease, membranoproliferative glomerulonephritis (MPGN) [55, 56]. These diseases are ultra-rare, affecting ~ 1 per million population [57–59].

### Classification and pathology of C3G

The classification of diseases in uncontrolled complement activation resulting in glomerulopathy continues to evolve. The term C3G was introduced to alert the clinician to the possibility of an underlying abnormality of the complement system [54]. C3G can be further classified by the pattern of dense deposits on electron microscopy. Dense deposit disease (DDD) is a specific form of C3G that is classified by the presence of dense osmiophilic intramembranous deposits seen on electron microscopy. C3 glomerulonephritis (C3GN) are forms of C3G in which deposits on electron microscopy may be light dense, amorphous mesangial, paramesangial, subendothelial, or subepithelial.

A diagnosis of C3G requires a renal biopsy. The presence of C3 dominant staining on immunofluorescence (IF), with an intensity of at least two orders of magnitude greater than any other immunoreactant (IgG, IgM, IgA, and C1q) provides the best sensitivity and specificity for C3G [60]. This current definition of C3G captures about 90% of cases of DDD, and possibly fewer cases of C3GN [60]. Once C3G has been diagnosed on renal biopsy, light microscopy then identifies diverse patterns of glomerular injury that include MPGN [54, 60], and detects additional features such as crescentic disease or markers of chronic disease such as interstitial fibrosis and tubular atrophy.

MPGN is a pattern of glomerular injury characterized by the presence of mesangial expansion, cellular proliferation, and double-contouring of the glomerular basement membrane [61]. The classification of MPGN was historically based upon the position of electron dense deposits relative to the glomerular basement membrane. Three types of MPGN were defined in this manner, type 1 (subendothelial), type 2 or DDD (intramembranous), and type 3 (subendothelial and subepithelial) [62]. Type 1 and type 3 MPGN typically stained positive for immunoglobulins and C3 on IF whereas type 2 MPGN typically stained for C3 only. MPGN of all types were previously associated with uncontrolled complement activation.

### Overlap of C3G and MPGN

**Fig. 2:**
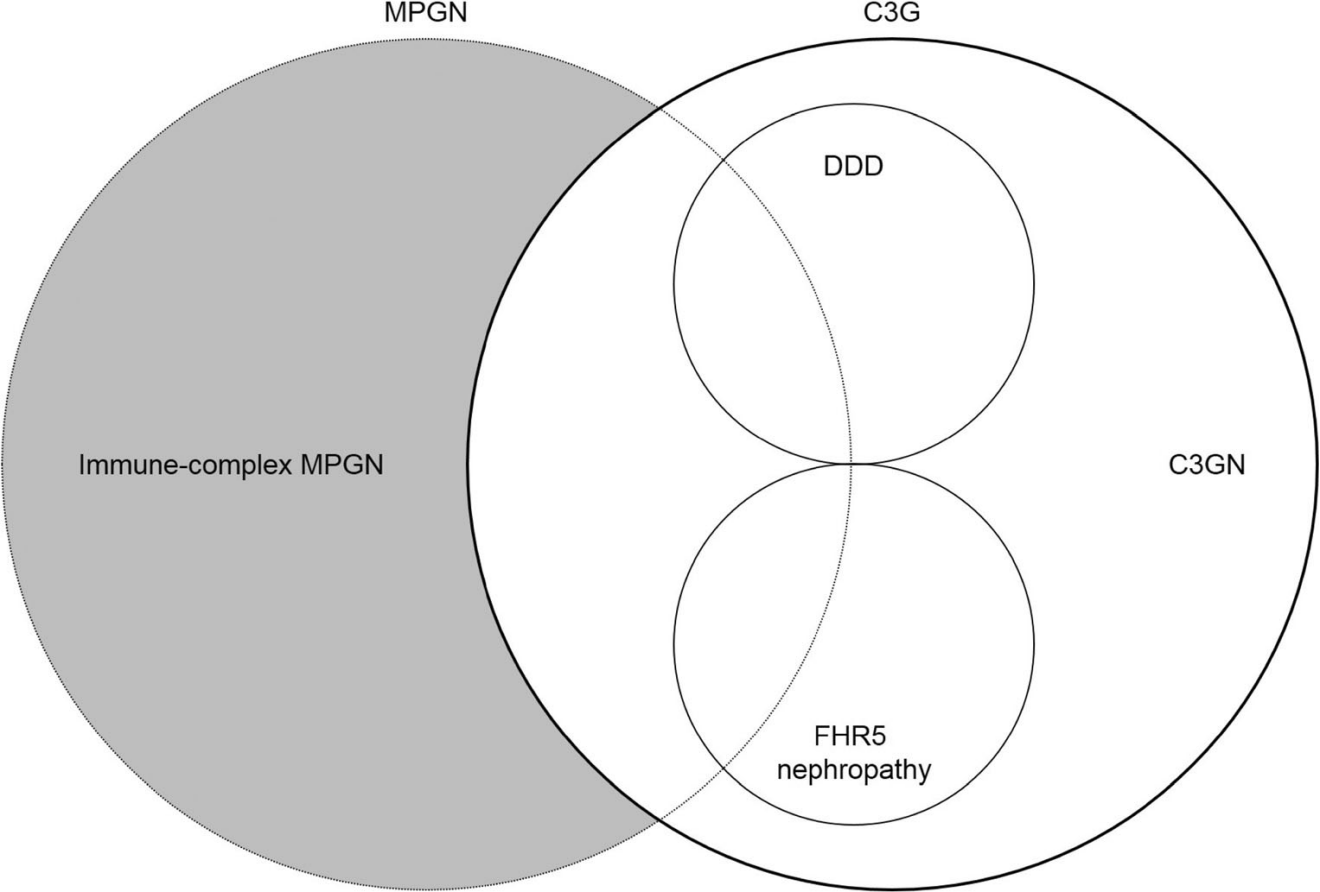
Overlap of C3G and MPGN. A cause of uncontrolled complement activation should be suspected in cases of C3 glomerulopathy (bold circle). Specific forms of C3G include C3GN, DDD, and CFHR5 nephropathy. Light microscopy identifies a diverse pattern of glomerular injury that includes MPGN. Uncontrolled complement activation has also been identified in cases of immune-complex MPGN (shaded)—these are cases of MPGN that do not fulfill current criteria for C3G. Causes of uncontrolled complement activation should be considered in an overlapping group of C3G and MPGN

The classifications of C3G and MPGN overlap [63]. The new classification of C3G includes cases of MPGN that were previously classified as type 1, 2, or 3. Furthermore, the definition of C3G identifies cases without MPGN, in which uncontrolled complement activation might not have been recognized [64]. Evidence of uncontrolled complement activation continues to be found in cases of MPGN that do not meet the diagnostic criteria of C3G [65–67]. Currently, these cases are termed immune-complex MPGN. Therefore, suspicion of uncontrolled complement activation should include the current definition of C3G and an overlap with cases of immune-complex MPGN (Fig. 2).

### Immune-mediated glomerulonephritis

Although uncontrolled complement activation may result in immune-complex MPGN, other secondary causes of MPGN should also be considered. These causes are summarized [68]. Treatment can then be directed at the secondary cause.

### Monoclonal gammopathy of renal significance

The absence of immunoglobulin on renal biopsy (in the setting of C3 deposition) is the hallmark of a diagnosis of C3G, usually indicative of an underlying disorder of complement regulation. However, it is increasingly recognized that deposition of monotypic immunoglobulin may be masked [69], and only identified following pronase digest. Upon identification of monoclonal deposits on the renal biopsy, the possibility of a monoclonal gammopathy needs to be considered. Treatment of these conditions is usually directed at the cause of the monoclonal gammopathy [70].

### Complement derangement in C3G and MPGN

A range of acquired and inherited abnormalities have been described as a cause of complement dysregulation in cases of C3G and MPGN. Unlike in aHUS, these abnormalities tend to result in fluid phase dysregulation of AP and acquired abnormalities predominate.

### FH deficiency

FH deficiency leads to uncontrolled activation of AP in the fluid phase [71, 72] and causes MPGN and C3G [56, 64, 73]. Genetic causes of FH deficiency are summarized in Table 2. Briefly, FH deficiency can be quantitative and due to mutations in *CFH*, inherited in homozygosity or heterozygosity, resulting in complete or partial FH deficiency, respectively. Other pathogenic mutations in *CFH* lead to a functional deficiency of FH due to the expression of proteins that have defective binding to C3b resulting in impairment of fluid phase complement regulation [80, 81, 83]. Overall, the prevalence of rare genetic variants in these cohorts ranges from 4 to 16.2% of patients [65, 66, 87]. The functional significance of a number of other rare genetic variants reported in these case series of MPGN or C3G is not known (Table 2). [96]

**Table 2:** Rare genetic variants in *CFH*, *CFI*, *CD46*, *C3*, and *CFB* reported in C3G and MPGN

Gene	Effect	Variant		Gene	Effect	Variant	
*CFH*	Complete FH deficiency (homozygous)	P88T^*^	[66, 74]	*CFH*	VUS (normal FH levels)	P26S	£
		R127L^*^	[73]			ΔG122-E128	[65]
		C431S	[65, 73]			D130N	[65]
		C597R	[65]			A161S	[65]
		P621Y	[75]			IVS11 + 5	[65]
		C673S	[73]			G334A	£
		Y899X	[76]			G650V	[64], ^£^
		Y1008X	[66]			F717L	[65]
		W1096R^*^	£			H878Y	2
	Partial FH deficiency	P76X	[64]			A892V	3
		L77X	[65]			R1210V	[65]
		V143I	[65]		VUS (FH levels not known)	R127C	[66]
		I216T	[77]			S199G/E1172X	£
		R232X	[65], ^£^			C431S	£
		C673R	[65]			N516K	[78]
		K768X	[76]			V609I	£
		C1043X	[65]			M725X	[79]
	Functional FH deficiency (homozygous)	R78G	[66]			V837I/E1145D	[78]
		ΔK224^*^	[80]			Q950H	[66]
		R53C	[65, 81]			T956M	[78] [82] [66]
	Functional FH deficiency	R83S^*^	[83]	*C3*	Gain of function	Δ923-924DG^*^	[84]
		R1210C	[64] [66]			I756T^*^	[85]
		R53C	[65] [86]			R161W	[87]
*CD46*	VUS	K66N	[66, 88]		VUS	R148Q	[87]
		V181M	[65]			A443S	[87]
*CFI*	FI deficiency	G119R	[65, 89–91]			L1100P	[87]
		A240G	[65, 92, 93]			L1318R	[87]
		C309R	[65]			V86I	[66]
		C327R	[65]			R505C	[66]
	VUS	c.1-4C > T	[66]			V619M	[66]
		G57D	[66]			G637R	[66]
	None	G261D	[65, 79, 91, 94]			R1042Q	[66]
		I306S	[65]			S1063N	[66]
*CFB*	Gain of function	I242L	[87]			R1303H	[66]
	VUS	D279E	[87]			R1320Q	[66]
		S367R^*^	[95]			D1362N	[66]
		G161R	[66]			C1518R	[66]
		H451R	[66]			D1625H	[66]
		R679W	[66]		None	K1051M	[66]

All variants heterozygous except where indicated

*VUS* variant of uncertain significance, *FH* complement factor H, *FI* complement factor I. * reported in familial disease, £ unpublished, Δ amino acid deletion

### FHR proteins

**Fig. 3:**
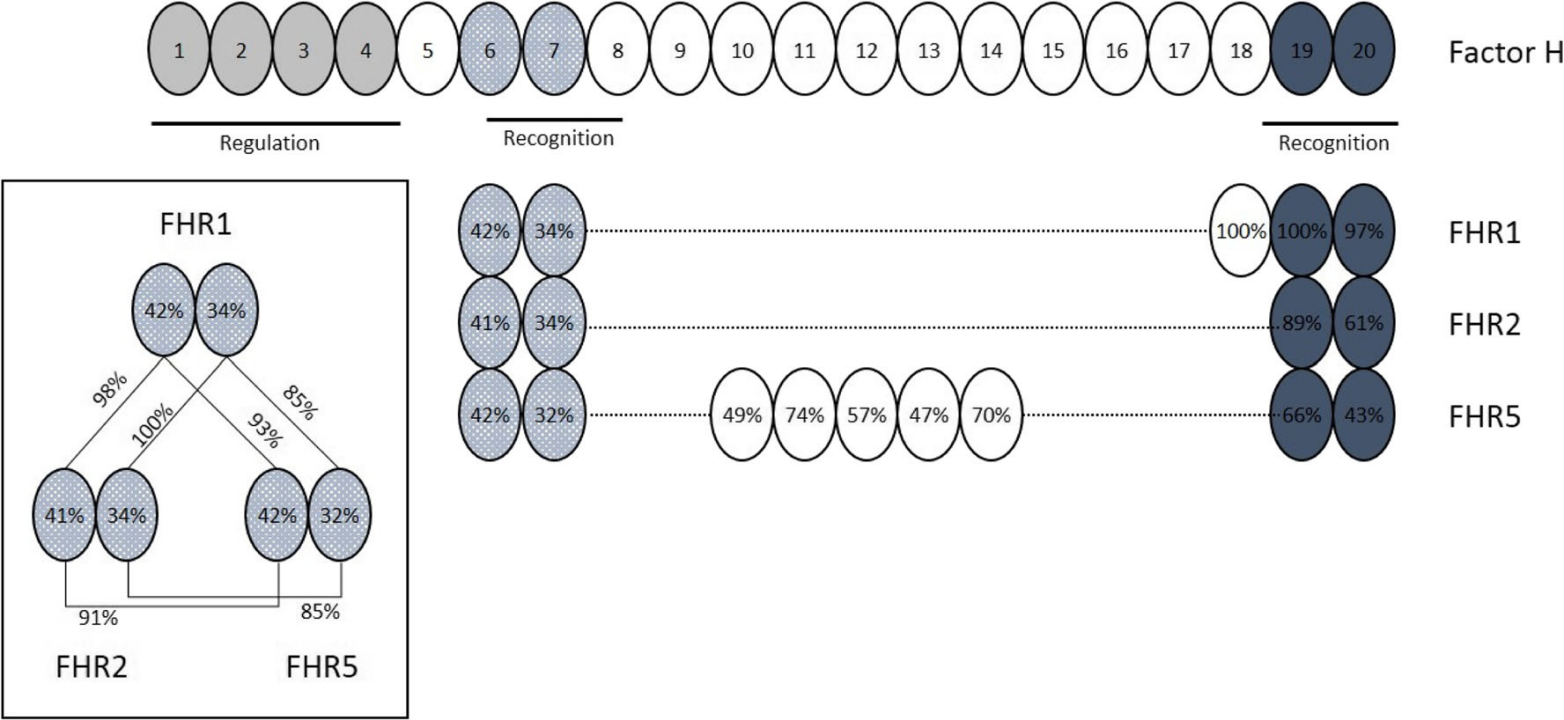
FHR1, FHR2, and FHR5 have a dimerization motif but lack regulatory domains. Shaded ovals denote regulatory and recognition domains of FH. Percentages shown within ovals of FHR proteins indicate degree of shared homology with corresponding SCR of FH depicted directly above. FHR proteins do not have shared homology with the regulatory domains of FH. However, SCR1 and 2 of FHR1, FHR2, and FHR5 (patterned ovals) have a high degree of shared homology with each other—highlighted in boxed inset. These domains share a dimerization motif

Recent studies suggest an important role of FHR proteins in complement regulation. FHR proteins may compete with FH for C3b binding and prevent the regulatory activities of FH on surface-bound C3b [97]. SCR1 and 2 of FHR1, 2, and 5 have a very high degree of sequence homology (Fig. 3) and share a dimerization motif. These studies show that these FHR proteins exist in dimeric form. FHR proteins do not have complement regulatory domains and as a result, dimeric forms of FHRs with high avidity to C3b act as competitive antagonists, preventing the normal regulatory function of FH and therefore “deregulating FH” [97].

### Genomic abnormalities in C3G

First identified in a large Cypriot pedigree [98], a number of genomic abnormalities in the RCA cluster have been described in familial cases of C3G (Table 3). In all cases, the genomic abnormality resulted in the formation of a larger *CFHR* gene resulting in FHR proteins with additional SCRs [97–105]. Functional study of dimeric forms of abnormal FHR proteins demonstrated an enhanced ability to compete with FH resulting in enhanced deregulation of FH as a possible mechanism of disease [97, 102]. In the case of the FHR2_-_FHR5 hybrid protein reported by Chen et al., functional studies showed evidence of stabilization of C3bBb in the presence of the hybrid FHR2_-_FHR5 protein resulting in complement activation in the fluid phase [99] and a possible role in binding properdin [100].

**Table 3:** Abnormal FHR proteins described in C3G

Abnormal FHR protein	Phenotype	Effect	Reference
FHR2_12_FHR5_1–9_	DDD	Stabilizes C3bBb	[99, 100]
FHR5_12_FHR5_1–9_	*CFHR5* nephropathy	De-regulates FH	[97, 98]
FHR5_12_FHR2_1–4_	C3GN	Not known	[101]
FHR1_1234_FHR1_1–5_	Low C3	De-regulates FH	[102]
FHR3_12_FHR1_1–5_	C3GN	De-regulates FH	[97, 103]
FHR1_123_FHR5_1–9_	DDD/C3GN overlap	De-regulates FH	[104]
FHR5_12_FHR2_1–4_	C3GN	De-regulates FH	[105]

Abnormal FHR protein—the subscript indicates the SCR of each FHR protein that form the abnormal FHR protein

*FHR* factor H-related, *DDD* dense deposit disease, *C3GN* C3 glomerulonephritis, *C3bBb* C3 convertase of the alternative pathway, *FH* factor H

Rare genetic variants in *CFHR5* have also been reported in C3GN and include C269R [106] and N216P [79]. The functional significance of both variants has yet to be determined.

### Complement factor I and membrane cofactor

Rare genetic variants in other complement regulators (*CFI* and *CD46*) are infrequently identified in C3G and MPGN (Table 2). Reported variants have all been inherited in heterozygosity. A few (resulting in low FI levels [65, 89, 90]) are pathogenic but their importance in disease pathogenesis has not been established.

### Components of the AP C3 convertase—C3 and FB

Detailed functional studies of several familial rare genetic variants in *C3* in C3G have been described. In a case of familial DDD, the variant Δ923-924DG does not undergo conformational change to C3b but does form a C3 convertase that is resistant to decay by FH [84]. In a report of familial C3GN, the variant I756T results in defective C3b inactivation by FI in setting of cofactors CR1 and FH [85].

Rare genetic variants in *C3* and *CFB* have also been described in cohorts of C3G and MPGN [87] [66] and includes the S367R variant in *CFB* in one familial case of C3GN [95]. Most of these variants have not been functionally studied (Table 2).

### Polymorphisms

Common genetic susceptibility factors have been reported in MPGN and C3G. In DDD, the Y402H polymorphism in *CFH* [65, 107, 108] associates with an increased risk of disease. Conversely, the V62I polymorphism was shown to be protective against DDD [26, 66].

Risk haplotypes in *CFH* also associate with disease. The H1 haplotype that carries the at-risk Y402H SNP in DDD associates with an increased risk of DDD [26, 107]. The H2 haplotype that carries the protective SNP V62I was shown to be protective in DDD [26].

Common SNPs and haplotypes in *CD46* have been studied in cohorts of C3G and MPGN. The intronic SNP c.-652G was protective in MPGN and C3GN. This association was also observed in cases of MPGN type 1 and C3GN, in which the haplotype *CD46*_AAGGT_ was observed more frequently, while the haplotype *CD46*_GAGGT_ was observed less frequently [65]. These findings were not observed in a later study [66]. In this later study, the SNPs c.-366A>G and c. *783 T>C were observed more frequently in immune-complex MPGN than controls [66]. In the same later study, the SNP c.-366A was observed more frequently in DDD compared to controls [66].

Several common SNPs in *C3* and CFB have been studied in C3G and MPGN. The SNPs, R102G [108, 109], and P314L in *C3* are associated with DDD [108]. The SNPs in *CFB*, R32W, and R32Q were not associated with C3G or MPGN [66].

### Acquired abnormalities in C3G and MPGN

The first acquired abnormality discovered in MPGN was a circulating factor in serum that was found to increase cell lysis in the fluid phase [55]. This was later discovered to be IgG that stabilized C3bBb by 10-fold [110, 111] now known as C3 nephritic factor. There is a strong association of C3 nephritic factor with all forms of C3G and MPGN, and is prevalent in up to 80% of DDD and 50% of MPGN and C3GN [65, 78]. C3 nephritic factors are not specific to MPGN and C3G and have been observed in acquired partial lipodystrophy [112] and in normal individuals [113].

Autoantibodies to individual complement components and their regulators have been reported in cases of C3G and MPGN. Autoantibodies to FB were first described in DDD [78, 114]. In one patient, these were shown to stabilize C3bBb, causing C3 consumption and terminal pathway activation [114]. No functional data are available in a further report in which three DDD patients with autoantibodies to FB are described [78]. Autoantibodies to C3b and FB in the same patient were described in two cases of DDD. These patients lacked C3 nephritic factor but the antibodies enhanced C3bBb activity [115]. Autoantibodies in FB and C3b have recently been reported in a cohort of C3G and MPGN [67]. Functional studies of purified IgG from patients in this cohort also result in AP activation.

Autoantibodies to FH have also been described in patients with C3G and MPGN. These bind predominantly to the N-terminal domain of FH and impair the regulatory activity of FH [78, 116]. The prevalence of autoantibodies to FH was 11% in a MPGN/C3G cohort [117]. In this cohort, patients with autoantibodies to FH did not associate with homozygous deletion of *CFHR3*/*1*. These autoantibodies associated with C3 nephritic factor in children and monoclonal gammopathy in adults [117]. The association of autoantibodies to FH and monoclonal gammopathy had been previously described in case reports [118, 119].

### Clinical presentation and outcome of C3G and MPGN

MPGN and C3G, especially the subset of patients with DDD, are typically diseases of childhood and young adulthood [57, 65]. They present with similar clinical features that include proteinuria, hematuria, and renal failure [57, 65]. Renal failure is progressive and 40% of patients develop end-stage renal disease (ESRD) at 10 years [57, 65, 120]. Recurrence in transplantation is common in all types, ranging from 30 to 40% in MPGN type 1 to 80–90% in DDD [121]. Plasma C3 levels are often low [57, 65, 66, 122]. Individual complement abnormalities have not been associated with a greater risk of adverse outcomes [57, 65] although the absence of a C3 nephritic factor or a rare genetic variant did have a higher risk of progression to ESRD in an Italian cohort [66].

There is an association of C3G and MPGN with extra-renal manifestations: acquired partial lipodystrophy [123, 124] and drusen [125].

The clinical features of patients with FHR5 nephropathy, a subset of patients with C3G, differ from other forms of C3G. First reported in large pedigrees with Cypriot ancestry, these patients often have Synpharyngitic hematuria. Prognosis is often worse in males with FHR5 nephropathy [98].

### Treatments in C3G and MPGN

There are no universally effective treatments for C3G or MPGN. The only double blind randomized control trial in this group of patients was performed in 1992 [126]. Eighty children (with MPGN types 1, 2, and 3) were randomized to receive 40 mg/m^2^ of prednisolone on alternate days. In this study, long-term treatment with prednisolone did appear to improve the outcome of patients with MPGN. Other studies suggest some benefit from the use of cyclophosphamide [127], mycophenolate mofetil (MMF) [128, 129] and the combination of aspirin and dipyridamole [130–132]. The use of rituximab has been described in case reports only [133–135].

### Complement inhibition

Unlike in aHUS, the role of complement inhibition in C3G and MPGN is not clear, despite the role of uncontrolled complement activation in disease. To date, there is one clinical trial of eculizumab in MPGN that is currently underway [136]. Nonetheless, eculizumab use has been reported in a growing number of cases of C3G and MPGN. Current reports suggest a more beneficial role for eculizumab in cases where prominent terminal pathway activity is seen [137–141].

Other reported therapies that modulate complement include the replacement of FH in patients with a functional deficiency of FH using plasma exchange [142] and soluble CR1 [143]. Further studies are required to determine whether complement inhibition in C3G can be effective.

## Paroxysmal nocturnal hemoglobinuria

Paroxysmal nocturnal hemoglobinuria (PNH) is a rare hemolytic anemia first described in 1882 [144]. In addition to hemolysis, thrombosis, muscle dystonias, chronic kidney disease, and bone marrow failure may occur [145]. The incidence of PNH is ~ 1–1.5 cases per million individuals worldwide [146]. Most patients present between the ages of 30–59 [146] and it is rare in children [147].

### Genetics

Unlike the germline mutations described commonly in aHUS and less commonly in C3G, PNH is caused by somatic mutations in the phosphatidylinositol glycan anchor biosynthesis class A gene (*PIGA*) in one or more long-lasting hematopoietic stem cell (HSC) clones [148–150]. The majority of mutations seen are indels resulting in frameshifts [151, 152]. *PIGA’s* location on the X chromosome accounts for the ability of one somatic mutation to cause PNH as only one allele is functional in men and women.

The expansion of *PIGA*-deficient HSC clones is central to the clinical phenotype of PNH; however, the *PIGA* mutations do not themselves confer a growth advantage [153, 154]. Both intrinsic (additive genetic and epigenetic variations [155]) and extrinsic (bone marrow failure [156]) clonal mechanisms have been suggested to account for the clonal expansion (reviewed [145]).

Although there are multiple genes involved in GPI synthesis, there is only one case of PNH reported where *PIGA* mutations were not seen. In this case, one somatic mutation in addition to a germline mutation in *PIGT* caused lack of GPI anchored cells and the PNH phenotype [157, 158].

### Pathogenesis

*PIGA* encodes for a glycosyl transferase that is required in the biosynthetic pathway for the synthesis of glycosyl phosphatidylinositol (GPI) [159]. *PIGA* mutations lead to a deficiency of GPI-anchored proteins including CD14, CD16b, CD48, and the complement inhibitor proteins CD55 (decay accelerating factor; DAF) and CD59.

CD55 accelerates the decay of the alternative and classical pathway C3 and C5 convertases while CD59 is a terminal pathway regulator which binds C8 preventing C9 recruitment and formation of the membrane attack complex. Deficiency of these complement regulators is critical to PNH erythrocytes being susceptible to complement-mediated attack.

In addition to the dominant hemolytic clinical feature of PNH, thrombosis is commonly seen. The exact mechanisms of thrombosis in PNH are unclear (reviewed by Hill et al. [160]) although the interplay of the complement and coagulation cascades, particularly C5 receptor signaling pathways, activation of platelets and intravascular hemolysis have been suggested.

### Clinical presentation and outcome

In PNH-affected erythrocytes, the constant activation of the AP causes a chronic low-level hemolysis and at time of infections or other complement triggering events there may be a hemolytic attack [161]. The most common cause of mortality of PNH is thromboembolism, with venous thrombosis more common than arterial thrombosis [160]. Smooth muscle dystonias including back pain, abdominal pain, erectile dysfunction, and dysphagia are seen [162]. Chronic kidney disease can occur with hemosiderin deposition leading to tubulointerstitial inflammation [163, 164]. Bone marrow failure is commonly seen in PNH but is not a consequence of the somatic mutations in *PIGA* [145].

Historically, the median survival of patients with PNH was approximately 10 years [165–167]; however, since the introduction of eculizumab, in those without bone marrow failure, a normal lifespan can be expected [146, 168].

### Eculizumab in PNH

Eculizumab is currently the only licensed therapy for PNH. Its introduction resulted in improvement in intravascular hemolysis [169, 170], thrombosis [171], renal function [172], and survival [168]. Variable response to treatment is seen with some patients presenting with residual hemolysis and requiring red blood cell transfusions. Low-level extravascular hemolysis is seen in most PNH patients on eculizumab. In this setting, C3 fragments opsonizing erythrocytes are recognized by macrophages in the spleen and liver resulting in their destruction [173]. It has recently been shown that a polymorphism in *Complement receptor 1* (*CR1*) alters the level of CR1 on erythrocytes and consequently the level of C3 opsonization of erythrocytes [174]. This increases the clearance of the erythrocytes.

As seen in aHUS, a polymorphism in *C5* can limit eculizumab’s effectiveness and complete blockage should be confirmed (AH50/CH50/sC5b-9) [52].

### Novel complement inhibitors in PNH

Several novel complement inhibitors are in various stages of development for the treatment of PNH including the C5 inhibitors ALXN1210 (Alexion; NCT02946463, NCT03056040), RA101495 (RaPharma NCT03030183, NCT03078582), ALNCC5 (NCT02352493 Alnylam), Coversin (NCT02591862; Akari), the C3 inhibitor compstatin analogue APL-2 (NCT02588833; Apellis), and the factor D inhibitor ACH-4471 (Achillion). These are reviewed in more detail in this issue by Harris [175].

### Summary

Together, these classical diseases of complement dysregulation provide a window on the vastly different phenotypes that can result from the subtle variations in complement regulation. These diseases will provide the test bed for the next generation of complement inhibitory agents.
